# Silencing of O-linked N-acetylglucosamine transferase ameliorates hypercalcemia-induced neurotoxicity in renal failure by regulating EZH2/KLF2/CXCL1 axis

**DOI:** 10.1038/s41419-021-04022-x

**Published:** 2021-08-30

**Authors:** Yaochen Cao, Xin Chen, Hongming Sun

**Affiliations:** 1 Department of Nephrology, Charité - Universitätsmedizin Berlin, Campus Mitte, Berlin, Germany; 2 Department of Neurology, the Fourth Hospital of Daqing, Daqing, P. R. China

**Keywords:** Neuroscience, Kidney diseases

## Abstract

Hypocalcemia, associated with Calcium neurotoxicity, has been reported to induce nerve dysfunction, which is a significant problem of renal failure. This study identifies a molecular mechanism of the O-linked N-acetylglucosamine transferase (OGT)-mediated enhancer of zeste homolog 2 (EZH2)/krüppel-like factor 2 (KLF2)/chemokine (C-X-C motif) ligand 1 (CXCL1) axis underlying the hypercalcemia-induced nerve injury in renal failure. Bioinformatics analyses were used to screen out the key factors in hypercalcemia-induced nerve injury in renal failure. Chronic kidney disease (CKD) was induced by an adenine diet in mice, followed by injection of adenovirus vector carrying short hairpin RNA targeting OGT, followed by behavioral tests and collection of the cerebral cortex for primary neurons. Calcium level in neurons was measured by Fluo-4-am and Perkin Elmer+ Operetta. Neuronal apoptosis and viability were detected by flow cytometry and the MTS method. The binding of EZH2 to KLF2 promoter was verified by chromatin immunoprecipitation assay. The concentration of Ca^2+^ in brain tissues of CKD model mice was increased, and nerve functions were obviously damaged. High expression of OGT occurred in kidney tissue of CKD model mice. Silencing OGT reduced the hypercalcemia-induced toxicity of neurons by inhibiting the expression of EZH2, which elevated the expression of CXCL1 in primary neurons by diminishing KLF2. Silencing OGT attenuated hypercalcemia-induced neurotoxicity by regulating the EZH2/KLF2/CXCL1 axis. In vivo experiments further confirmed that silencing OGT could reduce hypercalcemia-induced nerve injury in CKD mice. Taken together, silencing OGT downregulates EZH2, which increases the expression of KLF2 and then decreases the expression of CXCL1, thus alleviating hypercalcemia-induced nerve injury in renal failure.

## Introduction

Renal failure is regarded as a considerable cause of disability and mortality, and a typical feature of this disorder and its therapy dialysis is encephalopathy [1]. If developing to the end stage, chronic renal failure will manifest complete loss of kidney function [2]. Of note, severe hypercalcemia may rarely occur during renal failure and cause morbidity and mortality [3]. To our acknowledge, hypercalcemia can lead to neuropsychiatric dysfunction such as mood and cognitive changes, and high calcium levels can serve as a catalyst for neuronal demise, probably because of glutaminergic excitotoxicity as well as dopaminergic and serotonergic dysfunction [4].

O-linked N-acetylglucosamine transferase (OGT) is identified as an important enzyme that is able to catalyze the covalent bonding of N-acetylglucosamine in the target protein to the hydroxyl group of either serine or threonine [5]. Of note, O-GlcNAc plays a crucial role in the development of diabetes with complications that can lead to renal failure and nerve damage [6]. Intriguingly, it was found that OGT could regulate EZH2/histone H3 lysine 27 trimethylation (H3K27me3) in dorsal area CA1 of the hippocampus in the course of memory consolidation [7]. Enhancer of zeste homolog 2 (EZH2) is a well-recognized methyltransferase that can mediate H3K27me3 and exert an important role in multiple kidney diseases [8]. EZH2 was revealed to regulate the spinal neuroinflammation in the spinal dorsal horn in rats with neuropathic pain post nerve injuries [9]. EZH2 could result in a diminished level of Krüppel-like factor 2 (KLF2) in multiple cancers such as cervical cancer [10] and non-small cell lung cancer [11]. KLF2 is recognized as a type of transcription factor that can regulate the levels of chemokine receptors [12]. Intriguingly, it was found that suppression of KLF2 was a consequence of the uremic milieu and could further exacerbate endothelial dysfunction as well as cardiovascular diseases [13]. According to a previous study, KLF2-deficient macrophages displayed increased expression of chemokine (C-X-C motif) ligand 1 (CXCL1) [14]. Upregulated CXCL1 promoted renal ischemia-reperfusion injury, which can cause intrinsic acute renal failure [15] Considering all the above findings, we hypothesize in this study that OGT may affect hypercalcemia-induced nerve injury in renal failure by regulating the EZH2/KLF2/CXCL1 axis.

## Materials and methods

### Ethical approval

The present study was performed with the approval of the Ethics Committee of the Fourth Hospital of Daqing. All experimental procedures obeyed the Guidelines for the Care and Use of Laboratory Animals issued by the National Institutes of Health.

### Experimental animals

A total of 32 adult male C57/BL6 mice (8-weeks-old, 25–30 g) were purchased from the Hunan SJA Laboratory Animals Co., Ltd., Changsha, Hunan, China. Under standard environmental conditions (temperature: 22–25 °C, humidity: 45−50%, and light/dark cycle each for 12 h), all animals were raised separately, and the standard diet was maintained before chronic kidney disease (CKD) induction, and they were free to eat and drink.

### Establishment and treatment of CKD model in C57/BL6 mice

Mice were assigned into 2 groups on a random basis, the control group (*n* = 8, on a standard diet) and the CKD group (*n* = 24). We used an adenine diet to induce CKD in a mouse model. Mice were fed with casein-rich food for 7 days, then 0.2% adenine was added to the diet for 7 days to induce renal tubular injury. Thereafter, the mice were maintained on a 0.15% adenine diet.

Then, 16 CKD mice were randomly selected, 8 of which were treated with negative controls (NC) for short hairpin RNA (shRNA) (the sh-NC group), while the remaining 8 mice were treated with shRNA against OGT (the sh-OGT group). sh-OGT adenovirus was constructed and packaged by Genechem (Shanghai, China). The recombinant adenovirus vector expressing green fluorescent protein and diluted to 5 × 10^5^ μL with phosphate-buffered saline (PBS). On the 7th day after the establishment of the model, 200 μL adenovirus carrying sh-NC and sh-OGT were injected into the mouse brain at a three-dimensional point. At the end of the behavioral experiment, kidney samples were harvested for protein analysis and histological examination. The kidney and brain were fixed in 10% neutral formalin solution for 24 h, dehydrated by gradient alcohol, and permeabilized with xylene; the embedding tray was dried in the air and placed in a wax vat, and the tissues were taken out and embedded in the embedding bracket, that is, the wax blocks containing tissue blocks were made and sliced at a thickness of 4 μm. The remaining part was frozen in liquid nitrogen. Blood samples were taken from eyeballs for the determination of serum creatinine and uric acid.

### Detection of serum biochemical indexes

Serum creatinine (SCr) and blood urea nitrogen (BUN) levels were detected by an automatic biochemical analyzer.

### Measurement of calcium content

The brain tissues were rewarming slowly, 0.5 g from each group was added with 6–10 mL of mixed acid (HNO3: HCIO4 = 4:1). After cold digestion for 24 h, the brain tissues were heated and digested on the current plate. The mixed acid was added to the tissue dissolution solution and heated to produce more white smoke. The calcium content was analyzed by atomic absorption spectrophotometer.

The hippocampal neurons in the brain tissues were incubated with 5 μm Fluo4-AM at 37 °C for 40 m. After incubation, the cells were washed with D-hanks solution to remove extracellular Fluo4-AM. Fluorescence signals were detected by a laser confocal scanning system (LSM 880, Germany). Fluo4 at 488 nm was excited by an argon laser and the emission was measured at 530 nm. The fluorescence intensity was quantified by Harmony (PerkinElmer, Norwalk, Connecticut, USA).

### Behavioral experiment of mice

Morris water maze experiment (escape latency): the water maze was a circular pool (150 cm in diameter and 60 cm in height) at a temperature of 20–25 °C. The water maze was divided into four quadrants: lower right, upper right, lower left, and upper left. A platform was installed in the lower right quadrant. Firstly, mice were given a block of four trials for five daily sessions.

In the first 4 days, the platform was placed 1.5 cm below the water surface for the purpose of spatial learning assessment. The mouse was placed in the water face to face and the time (escape latency) to find the platform within 2 m was recorded. If the mouse found the platform within 2 m, the actual escape latency within 2 m was recorded. If the platform was not found within 2 m, the experimenter directed the mouse to the platform and allowed the mouse to stay for 10 s, and the escape latency was recorded as 2 m.

On the fifth day, the platform was moved above water level for a spatial probe test. A trial was terminated once the mouse reached the escape platform or when 90 s had elapsed. The path of each mouse during each trial was automatically recorded by a computerized system and then analyzed by computing several parameters. A water entry point was fixed and the time of mice staying in the original platform quadrant (i.e., the target quadrant) was recorded as residence time within 60 s, and the number of times that mice swam through the original platform position, i.e., the number of rings.

Step down test: mice were stimulated by an electro-optic conditioned reflex platform (Institute of Medicine, Chinese Academy of Medical Sciences). The mice were placed face to the pool wall in turn. The copper grid was laid on the bottom surface. The voltage was 36 v; a platform with a height of 4.5 cm and a diameter of 4.5 cm was set in the left front corner of each reflex box. After feeding for 5 days, the mice were put into the reaction box to adapt for 5 m, and then we electrified and recorded the time taken for the mice to jump onto the platform and stabilize for 10 s. For each mouse, the reaction time and the number of errors were recorded.

### Hematoxylin and eosin (HE) staining

The kidney sections (4 μm thick) were fixed with 10% neutral formalin solution for 24 h. Dehydrated by gradient alcohol, they were permeabilized with xylene. The embedded tissues were sliced, permeabilized with xylene, dehydrated with gradient alcohol, and treated with distilled water for 1 m. Then, sections were stained with hematoxylin for 3 m. Next, the slices were treated with 0.5% hydrochloric acid alcohol differentiation solution for 10 s, returned to blue for 10 m, and treated with eosin staining solution for 5 m. Afterward, the slices were subjected to conventional dehydration, permeabilization, and blockade with neutral gum. The sections were observed under an optical microscope (XP-330, Shanghai Bingyu Optical Instrument Co., Ltd., Shanghai, China).

### Immunofluorescence assay

The mice brain slices were dewaxed to water, washed with PBS for 3 m, detached with compound enzyme for 1 m at room temperature, immersed in 0.01 mol/L sodium citrate solution for 30 m, and then treated with 0.3% Triton. X 100 for 20 m at room temperature. The normal goat serum was used to seal the slices for 1 h. The sections were incubated with mouse primary antibodies of neuronal markers NSE, dephospho-Tau, and MAP2, and then with fluorescence coupled secondary antibody IgG (ab150081, 1: 500, Abcam). Then, 4’,6-diamidino-2-phenylindole was used to stain the nuclei. Sections were analyzed by a pathologist with Zeiss LSM 780 confocal microscope (Germany) in a blind manner. *n* = 8 for each group.

### Terminal deoxynucleotidyl transferase-mediated deoxyuridine triphosphate-biotin nick end labeling (TUNEL) staining

To determine apoptosis, TUNEL staining was performed according to the protocol provided by Roche molecular system (Branchburg, NJ, USA). In order to evaluate TUNEL quantitatively, the number of TUNEL positive neurons was counted, and the average ratio to each microscopic field was calculated and plotted.

### Reverse transcription-quantitative polymerase chain reaction (RT-qPCR)

The total RNA was extracted from kidney and brain tissues by Trizol (Takara, Dalian, China) and reversely transcribed into cDNA using reverse transcriptase (Takara). The prepared cDNA was diluted and stored at −20 °C. Fluorescent qPCR was performed according to the instructions of SYBR® Premix Ex TaqTM I I(RR820A, Takara). Real-time fluorescent qPCR was performed by the ABI7500 qPCR instrument (7500, ABI, Oyster Bay, NY, USA) (Supplementary table 1). Using 2 μg total RNA as template and glyceraldehyde-3-phosphate dehydrogenase (GAPDH) as internal reference primer, the relative transcription level of EZH2 was calculated by relative quantitative method (2^-△△Ct^ method)

### Western blot analysis

Radio Immunoprecipitation Assay containing 1% protease inhibitor and phosphorylase inhibitor (Beyotime, Shanghai, China) was used to lyse tissues and cells. After centrifugation, the supernatant was taken. The protein concentration was determined by Bicinchoninic Acid Kit (Beyotime). The protein samples were prepared by boiling in 8−12% sodium dodecyl sulfate-polyacrylamide gel electrophoresis and electrotransferred to a polyvinylidene fluoride membrane. Then, 5% skimmed milk was used to seal the samples at room temperature for 1 h, which were then incubated overnight with corresponding antibodies: OGT (1: 2000, ab177941), EZH2 (1: 2000, ab228697), KLF2 (1: 2000, ab104481), CXCL1 (1: 2000, ab86436), B-cell lymphoma-2 (Bcl-2) (1: 2000, ab182858), Bcl-2 associated protein X (Bax) (1: 5000, ab32503), caspase 3 (1: 2000, ab13847), cleaved-caspase3 (1: 2000, ab2302), and GAPDH (1: 5000, ab181602). The antibodies were all purchased from Abcam. The next day, the antibody was recovered, washed with Tris Buffered saline Tween for 3 times, 5 m each time, and incubated with 1: 100 diluted horseradish peroxidase-labeled goat anti-mouse against immunoglobulin G (IgG) (HA1003, Shanghai Yanhui Biotechnology Co., Ltd., Shanghai, China) for 1 h. The membrane was reacted with enhanced chemiluminescence solution (ECL808-25, Biomiga, USA) for 1 m at room temperature. The liquid was absorbed and developed in the gel imager. GAPDH was used as an internal parameter, and the ratio of the gray value of the target band to that of the internal reference band was used as the relative expression quantity of protein.

### Chromatin immunoprecipitation (ChIP)

ChIP analysis was performed according to the commercially available ChIP kit (17–610, Millipore, Billerica, MA, USA). In short, the cells were centrifuged at 4 °C at 20000 × g for 10 m. The supernatant containing chromatin complex was collected and 1/10 of the total volume was used as the input sample. The protein-G agarose beads and antibody to EZH2 (1: 500, ab228697, Abcam) in chromatin samples were incubated overnight at 4 °C. After washing, chromatin complexes are eluted and crosslinked after a fracture. The purified DNA was used as a template for PCR-specific analysis of transcription factor binding sites in the transcription region of KLF2 genes. PCR products were separated by agarose gel electrophoresis and then stained by red fluorescent dye. ImageJ software (National Institutes of Health, Bethesda, MA, USA) was used to analyze the fluorescence intensity.

### Extraction of primary neurons from mouse cerebral cortex

C57/BL6 neonatal mice born within 24 h were disinfected with 75% ethanol to collect the brains, from which hippocampal tissues were rapidly harvested through blunt separation. Meninges and blood vessels were carefully removed, followed by 2 washes in ice D-Hanks solution. The hippocampus was cut into cubes of 1 mm [3] on ice. After 3 h of collagenase treatment, neurons were isolated and cultured in Dulbecco’s modified Eagle’s medium/F12 + 2% B27 (engreen) in a 5% CO_2_ incubator at 37 °C. On the 10th day, neuron-specific enolase (NSE) detected by immunofluorescence was used to identify the purity of neurons (Fig. S1), followed by detection of dephospho-Tau and MAP2 for further verification (Fig. S2).

EZH2 and CXCL1 overexpression plasmids were purchased from GenePharma (Shanghai, China). The cells were inoculated in a six-well plate with a density of 3 × 10^5^/well. When the cell growth density reached 50−60%, the transfection could be carried out with a lipofectamine 2000 (Invitrogen, Carlsbad, CA, USA) kit. Every 250 μL opti-MEM (GIBCO, Carlsbad, CA, USA) medium was added with 6 µL NC for small interfering RNA (siRNA) (si-NC), siRNA against OGT (si-OGT), si-EZH2, si-KLF2, NC for overexpressed genes (oe-NC), plasmids overexpressing CXCL1 (oe-CXCL1), oe-EZH2, and 6 μL lipofectamine 2000. After standing at room temperature for 5 m, the two liquids were evenly mixed together. After standing for 20 m, the two liquids were dropped into the cell culture well and mixed over shaking. Then, the cells were put into the cell incubator at 37 °C containing 5% CO_2_ for further culture. After 8 h, the medium was renewed, and the cells were collected 48 h after transfection.

### Comet assay

The cells (5–10 mL) isolated from the brain were embedded in low melting point agarose (0.5%) and placed on a completely ground microscope slide covered with PBS buffer of 0.75% normal agarose (diluted in Ca^2+^ and Mg^2+^). The last layer was placed on top of 0.5% low melting point agarose. The slides were immersed in pyrolysis buffer solution (12.5 mol/L NaCl, 100 mmol/L Na_2_Ethylene Diamine Tetraacetic Acid [Na_2_EDTA] and 10 mmol/L Tris, pH 10.0, addition of freshly prepared 1% Triton X-100 and 10% dimethyl sulfoxide) for 1 h at 4 °C. The slides were then incubated in freshly prepared electrophoresis buffer EDTA (pH 13) for 20 m and exposed to 25 V/300 MA for 20 m, followed by neutralization of the alkali in 0.4 m Tris (pH 7.5) for 15 m. Finally, DNA was stained by adding ethidium bromide (30 μg/mL, 50 μL).

The slides were scored by blind analysis and analyzed immediately. Under a fluorescence microscope (BX51; Olympus, Tyoko, Japan) equipped with a green light excitation filter and a 590 nm barrier filter, the tailing rate was evaluated by calculating the tailing DNA in 200 cells in each sample. Next, 25 cells were randomly selected and photographed to measure the length of DNA migration. The extent of DNA damage was assessed by subtracting the nuclear diameter from the total length of the image.

### Flow cytometry

After 48 h of transfection, the cells were detached with trypsin without EDTA (Thermo Fisher Scientific, Rockford, IL, USA) and collected in a flow tube. Following centrifugation at 2200 ×g for 30 m, the supernatant was discarded. The cells were washed with precooled PBS for 3 times, and centrifuged at 2200 ×g for 20 m, and the supernatant was discarded. According to Annexin-V-Fluorescein 5-isothiocyanate (FITC) apoptosis detection kit (Sigma-Aldrich Chemical Company), Annexin-V-FITC and propidium iodide (PI) (50: 1: 2) were mixed into Annexin-V-FITC/PI staining solution. Following the addition of 100 μL dye solution and incubation at room temperature for 15 m, the cells were added with 1 mL 2-[4-(2-Hydroxyethyl)−1-piperazinyl]ethanesulfonicacid buffer solution (Thermo Fisher Scientific). The apoptosis was detected by flow cytometry at 488 nm.

### 3-(4,5-dimethylthiazol-2-yl)−5(3-carboxymethonyphenol)−2-(4-sulfophenyl)−2H-tetrazolium (MTS) assay

Cell viability was determined by MTS cell proliferation Kit (celltiter96 Aqueous solution cell proliferation assay, Promega Corporation, Madison, WI, USA). The cells were seeded in 96-well assay plates until used. The culture was removed from the incubator and washed with Dulbecco’s phosphate-buffered saline (BI). MTS reagent (20 μL) was piped into each well of the 96 well assay plate, which contained 100 μL cells in a culture medium. The plates were incubated at 37 °C for 1–4 h in a moist atmosphere with 5% CO_2_. The absorbance at 490 nm was recorded by a 96-well microplate.

### Statistical analysis

All the data in this study were analyzed with the use of the SPSS 21.0 statistical software (IBM, Armonk, NY, USA). Each experiment was repeated three times independently. The measurement data were expressed by mean ± standard deviation. Data between the two groups were compared by unpaired *t-*test. Data among multiple groups were compared by one-way analysis of variance (ANOVA), and those at different time points by repeated-measures ANOVA, followed by Tukey’s post hoc tests. *p* < 0.05 indicated a statistically significant difference.

## Results

### OGT was upregulated in the kidney tissues of CKD mice

**Fig. 1:**
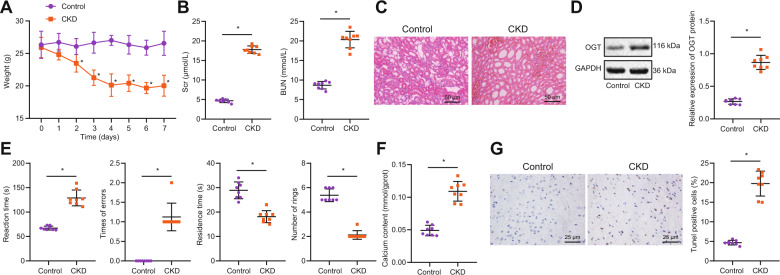
OGT is upregulated in the kidney of CKD mice. **A** Bodyweight of mice in each group from 0 to 7 days. **B** SCr, and BUN contents in serum. **C** HE staining for kidney tissue damage. **D** The protein expression of OGT in kidney tissues of each group detected by Western blot analysis. **E** Escape latency and step-down tests were used to observe the changes of nerve function in CKD mice. **F** the Calcium content in brain tissues of mice in each group. **G** TUNEL staining was used to detect neuronal apoptosis in brain tissues. *p < 0.05. vs. the control group (mice on a standard diet). *n* = 8. The experiment was repeated 3 times independently.

In order to investigate the effect of OGT on hypercalcemia-induced nerve injury induced by renal failure and its molecular mechanism, we first constructed a CKD mouse model. By measuring the weight changes of mice, we found that compared with the control group, the bodyweight of CKD mice decreased day by day, and finally tended to be stable (Fig. 1A). Serum biochemical results showed that SCr and BUN levels in CKD mice were higher than those in the control group (Fig. 1B). HE staining showed that the renal tissue structure of control mice was normal, the epithelial cells of renal tubules were intact, and there were no obvious pathological changes in glomeruli or the renal interstitium. Besides, renal tissues of CKD mice showed dilation of renal tubule lumen and hypertrophy of glomeruli, as well as edema and interstitial inflammatory infiltration of renal tubular epithelial cells (Fig. 1C). Western blot analysis showed that the expression of OGT in kidney tissues of CKD mice was higher than that in the control group (Fig. 1D). Compared with control mice, the learning and memory abilities of CKD mice were worse (Fig. 1E). Compared with the control group, the content of calcium in brain tissues of CKD mice was increased (Fig. 1F). TUNEL staining results showed that compared with the control group, CKD mice had more neuronal apoptosis (Fig. 1G). The above results indicate that calcium content in brain tissues of CKD model mice is increased and nerve function is obviously damaged. OGT is highly expressed in kidney tissues of CKD model mice.

### Silencing OGT reduced the toxicity of primary neurons induced by hypercalcemia

**Fig. 2:**
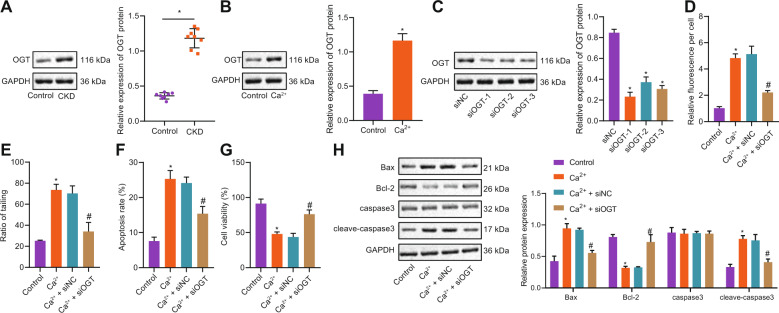
Silencing OGT reduces the toxicity of primary neurons induced by hypercalcemia. **A** Western blot analysis was used to detect the expression of OGT in brain tissues of mice in each group (n = 8). * *p* < 0.05. *vs*. the control group (mice on a standard diet). **B** Expression of OGT in neurons induced by hypercalcemia. *p < 0.05. vs. the control group (neurons cultured in basic medium). **C** Western blot analysis was used to detect the interference efficiency of three OGT interference sequences. *p < 0.05. vs. the si-NC group (neurons treated with si-NC). **D** luo-4-am, and Perkin Elmer Operetta were used to measure the intracellular calcium level. **E** Comet assay was used to observe the DNA damage of each group. **F** Flow cytometry was used to detect apoptosis. **G** MTS assay was used to detect the neuronal viability. **H** Western blot analysis was used to detect the expression of apoptosis-related proteins. *p < 0.05. vs. the control group (neurons cultured in basic medium). ^#^*p* < 0.05. vs. the Ca^2+^ + si-NC group (neurons treated with Ca^2+^ + si-NC). The experiment was repeated 3 times independently.

It has been confirmed that OGT is expressed in the kidney of CKD mice. Here, we further explore whether OGT can reduce hypercalcemia-induced neurotoxicity by silencing OGT. First of all, the expression of OGT in brain tissues of CKD mice was higher than that in the control group (Fig. 2A). After that, we primarily cultured mouse neurons, and the neurons were induced to produce hypercalcemia by using a high calcium concentration medium. The results showed that the expression of OGT in mouse neurons cultured in a high calcium environment was higher than that in the control group (Fig. 2B). Then, the expression of OGT in neurons was interfered with by transfection of si-OGT (Fig. 2C), and the best one was selected for an experiment. It was established that the concentration of Ca^2+^ in the neurons of mice cultured in a high calcium environment was increased. Besides, the Ca^2+^ concentration in mouse neurons was decreased in response to si-OGT (Fig. 2D). Comet assay results showed that the comet tail rate of neurons cultured in a high calcium environment was increased. Further, comet tail rate in Ca^2+^ + si-OGT-treated mice was decreased (Fig. 2E). Flow cytometric data showed that the apoptotic rate of neurons in a high calcium environment was increased, while the apoptotic rate of Ca^2+^ + si-OGT-treated mice was decreased (Fig. 2F). MTS results showed that the activity of neurons cultured in a high calcium environment was decreased, while the activity of neurons in Ca^2+^ + si-OGT-treated mice was increased (Fig. 2G). Western blot analysis showed that the expression of Bax and cleaved-caspase3 in a high calcium environment was increased along with reduced Bcl-2 expression while caspase3 expression did not differ. Moreover, downregulation of Bax and cleaved-caspase3 has been observed in Ca^2+^ + si-OGT-treated mice accompanied by upregulated Bcl-2 yet no significant difference was witnessed regarding caspase3 expression (Fig. 2H). These results suggest that silencing OGT can reduce the toxicity of primary neurons induced by hypercalcemia.

### Silencing OGT attenuated hypercalcemia-induced neurotoxicity by inhibiting EZH2 expression

**Fig. 3:**
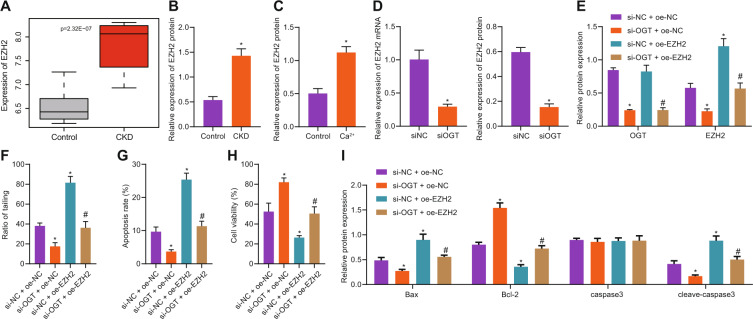
Silencing OGT attenuates hypercalcemia-induced neurotoxicity by inhibiting EZH2 expression. **A** The expression of EZH2 in CKD mice of the GSE148084 dataset. **B** The expression of EZH2 in brain tissues of CKD mice was detected by Western blot analysis (n = 8). * *p* < 0.05. *vs*. the control group (mice on a standard diet). **C** Western blot analysis was used to detect the expression of EZH2 in neurons induced by hypercalcemia. * *p* < 0.05. *vs*. the control group (neurons cultured in basic medium). **D** RT-qPCR, and Western blot analysis were used to detect the effect of OGT silencing on EZH2 expression. * *p* < 0.05. *vs*. neuron treated with si-NC. **E** Western blot analysis was used to detect the expression changes of OGT and EZH2 after simultaneous silencing of OGT and overexpression of EZH2. **F** Comet assay was used to observe the DNA damage of neurons in each group. **G** Flow cytometry was used to detect neuronal apoptosis. **H** MTS method was used to detect the vitality of neurons in each group. **I** The expression of apoptosis-related proteins was detected by Western blot. (**E**–**I**), *p < 0.05. vs. the si-NC + oe-NC group (neuron treated with si-NC + oe-NC). ^#^*p* < 0.05. vs. the si-OGT + oe-NC group (neuron treated with si-OGT + oe-NC). The experiment was repeated 3 times independently.

OGT has been reported to promote EZH2 expression by stabilizing OGT in breast cancer cells [16] while EZH2 has been indicated to facilitate CKD occurrence [17]. Hereby, it was inferred that OGT might mediate EZH2 in primary neurons induced by hypercalcemia. Based on the analysis of the CKD-related dataset GSE148084, we found that EZH2 was highly expressed in CKD (Fig. 3A). Western blot analysis showed that the expression of EZH2 in brain tissues and high calcium-induced neurons of CKD mice was higher than that of the control group (Fig. 3B, C). Then, OGT was silenced in primary neurons induced by hypercalcemia. RT-qPCR and Western blot analysis results showed that the expression of EZH2 mRNA and protein in the presence of si-OGT was decreased, indicating that silencing OGT could inhibit the expression of EZH2 in neurons (Fig. 3D). Subsequently, neurons were treated with silenced OGT or/and overexpressed EZH2. It was demonstrated that the protein expression of OGT and EZH2 in the presence of si-OGT was downregulated, and the protein expression of EZH2 in the presence of oe-EZH2 was upregulated where OGT protein level did not differ. Moreover, the expression of EZH2 in the presence of si-OGT + oe-EZH2 was upregulated (Fig. 3E). Comet assay was then performed to assess the degree of DNA damage, which was correlated with DNA migration distance (tail length) and DNA content (fluorescence intensity) distribution. The results showed that comet tail rate of neurons in presence of silencing OGT was decreased, and that in presence of overexpressed EZH2 was increased; when OGT was silenced and EZH2 was overexpressed at the same time, comet tail rate of neurons was increased (Fig. 3F). Flow cytometric data showed that the apoptotic rate of neurons in the presence of si-OGT was decreased, and that of the oe-EZH2 transduction was increased. Relative to si-OGT alone, the apoptotic rate of neurons in the presence of si-OGT + oe-EZH2 was increased (Fig. 3G). MTS for cell proliferation evaluation according to color depth showed silencing OGT enhanced the activity of neurons while overexpression of EZH2 suppressed neuron activity; additional EZH2 overexpression counterweighed the promoting action of silenced OGT on neuron activity (Fig. 3H). Expression of Bax, a pro-apoptotic protein, Bcl-2, an anti-apoptotic protein, and caspase3, a protein closely related to cell apoptosis, was then determined by Western blot analysis. It was found that in neurons treated with silenced OGT, expression of Bax and cleaved-caspase3 was reduced while Bcl-2 expression was elevated. Opposite results were observed in neurons treated with overexpressed EZH2. When OGT was silenced and EZH2 was overexpressed in neurons at the same time, Bax and cleaved-caspase3 expression was downregulated while Bcl-2 was upregulated. No significant difference was witnessed regarding caspase3 expression among groups (Fig. 3I). These results suggest that silencing OGT can reduce the toxicity of primary neurons induced by hypercalcemia by inhibiting the expression of EZH2.

### EZH2 promoted CXCL1 expression in primary neurons by inhibiting KLF2

**Fig. 4:**
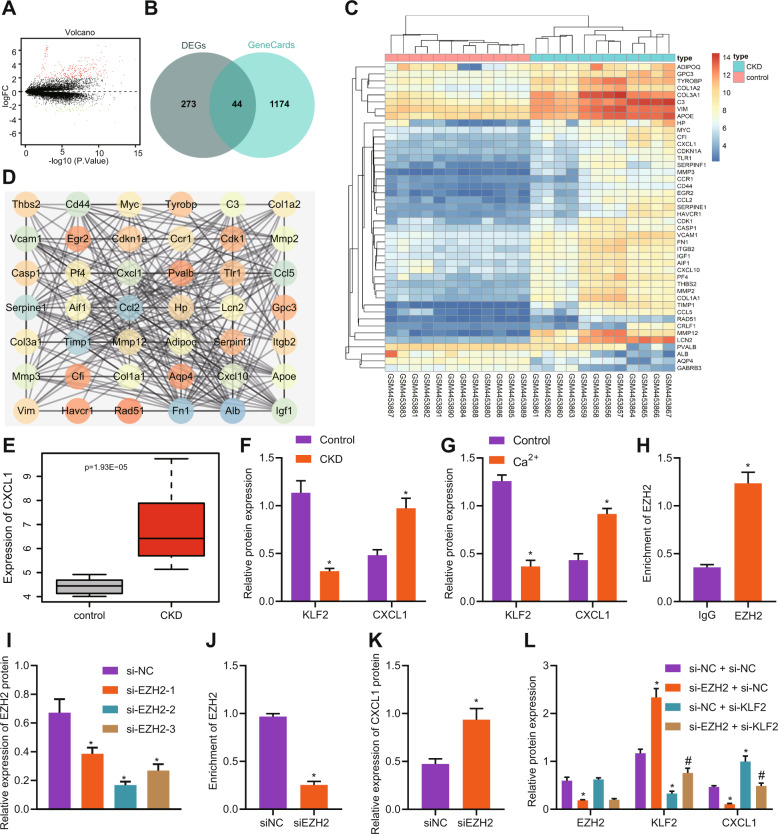
EZH2 promotes CXCL1 expression in primary neurons by inhibiting KLF2. **A** The differential expression in CKD model mice related dataset GSE148084 gene was analyzed by volcano map, and the abscissa showed −log10 (*p* Value), vertical coordinate represents log2FoldChange, the red dot indicates highly expressed gene, and green dot indicates poorly expressed gene. **B** Venn diagram of intersection of differential expression gene in CKD and nerve injury-related gene. **C** Differential expression heat map of a candidate gene in CKD dataset. The color scale from blue to red indicates expression value from small to large. **D** Gene interaction network diagram. The circle color scale from orange to blue indicates gene degree from low to high. **E** Expression of CXCL1 in CKD mice with GSE148084. F, The expression of KLF2 and CXCL1 in brain tissue of CKD mice was detected by Western blot analysis (*n* = 8). *p < 0.05. vs. the control group (mice on a standard diet). **G** Western blot was analysis used to detect the expression of KLF2 and CXCL1 in the neurons induced by hypercalcemia. *p < 0.05. vs. the control group (neurons cultured in basic medium). **H** The binding of EZH2 and KLF2 promoter was detected by ChIP assay. *p < 0.05. *vs*. IgG. **I** Western blot analysis on EZH2 interference sequence. *p < 0.05. *vs*. the si-NC group (neurons treated with si-NC). **J** ChIP detecting EZH2 concentration in the KLF2 promoter region. *p < 0.05. vs. the si-NC group (neurons treated with si-NC). **K** KLF2 protein expression was detected by Western blot analysis. *p < 0.05. vs. the si-NC group (neurons treated with si-NC). **L** Western blot analysis was used to detect the protein expression of CXCL1 after EZH2 and KLF2 silencing simultaneously. *p < 0.05. vs. the si-NC group (neurons treated with si-NC). ^#^*p* < 0.05. vs. the si-EZH2 + si-NC group (neurons treated with si-EZH2 + si^-^NC). The experiment was repeated 3 times independently.

By analyzing the gene differential expression of the GSE148084 in CKD model mice, 317 differentially expressed genes were obtained, including 269 highly expressed genes and 48 poorly expressed genes (Fig. 4A). Then, 1218 neural injury-related genes were screened from the GeneCards database (https://www.genecards.org/, Score > 10). The intersection of differentially expressed genes in CKD and nerve injury-related genes was extracted by jvenn online technology (http://jvenn.toulouse.inra.fr/app/example.html) to predict 44 key factors of hypercalcemia-induced nerve injury induced by renal failure (Fig. 4B), and the heat map of candidate genes in CKD-related dataset was drawn (Fig. 4C). The interaction network of candidate genes was further analyzed to obtain the gene interaction network (Fig. 4D). Among them, ALB, FN1, CCL2, TIMP1, serpine1, CCL5, CD44, VCAM1, IGF1, and CXCL1 were at the core of the network (degree > 18). Furthermore, it was found that CXCL1 was highly expressed in the pathogenesis of CKD. The expression of CXCL1 was significant in CKD-related dataset GSE148084 (Fig. 4E). In order to verify our hypothesis on whether EZH2 promoted CXCL1 expression in primary neurons by downregulating KLF2, we first detected the protein expression of KLF2 and CXCL1 in brain tissues and hypercalcemia-induced neurons of CKD mice. The results showed that KLF2 expression in brain tissues and hypercalcemia-induced neurons of CKD mice was decreased, and CXCL1 expression was increased (Fig. 4F, G). Microarray detection results showed that compared with the IgG group, the EZH2 group had more EZH2 on KLF2 promoter (Fig. 4H). Then, we screened the si-EZH2-2 sequence with the best interference efficiency by Western blot analysis for subsequent experiments (Fig. 4I). Microarray detection showed that EZH2 enriched on the KLF2 promoter in the presence of si-EZH2 was reduced (Fig. 4J). Western blot analysis showed that the expression of KLF2 protein in the presence of si-EZH2 was elevated (Fig. 4K). Next, Western blot analysis showed that the expression of EZH2 and CXCL1 in presence of si-EZH2 was downregulated, and the expression of KLF2 was upregulated; while the expression of KLF2 in response to si-EZH2 + si-KLF2 was decreased, and the expression of CXCL1 was increased (Fig. 4L). These results suggest that EZH2 can promote the expression of CXCL1 in neurons by inhibiting KLF2.

### Silencing OGT attenuated hypercalcemia-induced neurotoxicity by regulating the EZH2/KLF2/CXCL1 axis

**Fig. 5:**
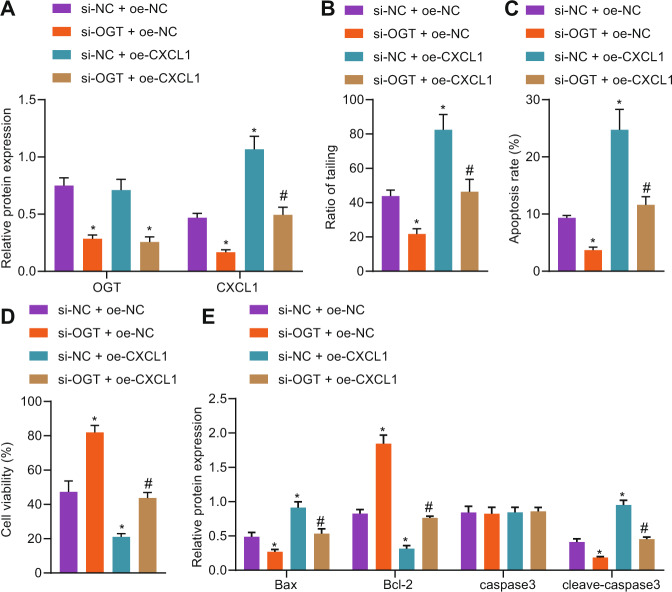
Silencing OGT attenuates hypercalcemia-induced neurotoxicity by regulating the EZH2/KLF2/CXCL1 axis. **A** Western blot analysis was used to detect the expression of OGT and CXCL1. **B** Comet assay was used to observe DNA damage. **C** Flow cytometry was used to detect apoptosis. **D** MTS assay was used to detect neuronal viability. **E** Western blot analysis was used to detect the expression of apoptosis-related proteins. *p < 0.05. vs. the si-NC + oe-NC group (neurons treated with si-NC + oe-NC). ^#^*p* < 0.05. vs. the si-OGT + oe-NC group (neurons treated with si-OGT + oe-NC). The experiment was repeated 3 times independently.

Next, OGT silencing and CXCL1 overexpression were performed simultaneously in hypercalcemia-treated mouse neurons to further verify the effect of the OGT/EZH2/KLF2/CXCL1 axis on hypercalcemia-induced neurotoxicity. Western blot analysis showed that the expression of OGT and CXCL1 in the presence of si-OGT was downregulated, CXCL1 expression in the presence of oe-CXCL1 was upregulated, compared with the si-OGT alone, CXCL1 expression in the presence of si-OGT + oe-CXCL1 was upregulated (Fig. 5A). According to results of comet assay (Fig. 5B), flow cytometry (Fig. 5C), MTS assay (Fig. 5D), and Western blot analysis (Fig. 5E), comet tail rate and apoptosis rate of neurons treated with si-OGT were lower along with enhanced neuron activity accompanied by downregulation of Bax and cleaved-caspase3 as well as upregulation of Bcl-2. Overexpression of CXCL1 led to opposite changing tendency. Also, lower comet tail rate and apoptosis rate of neurons as well as enhanced neuron activity were observed when OGT was silenced and EZH2 was overexpressed at the same time. However, caspase3 expression did not differ among groups. These results suggest that silencing OGT can alleviate hypercalcemia-induced neurotoxicity by regulating the EZH2/KLF2/CXCL1 axis.

### Silencing OGT in vivo attenuated hypercalcemia-induced nerve injury in CKD mice

**Fig. 6:**
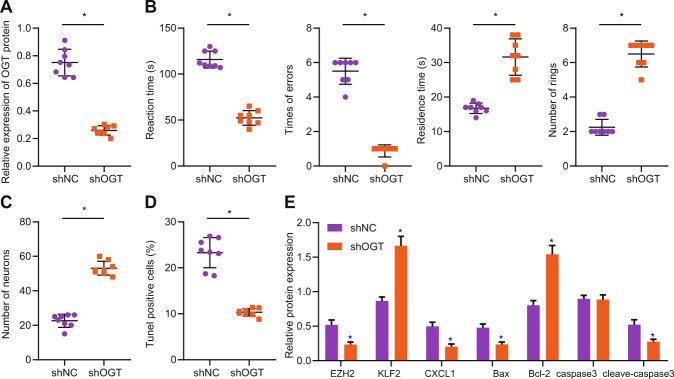
Silencing OGT in vivo attenuates hypercalcemia-induced nerve injury in CKD mice. **A** Western blot analysis was used to detect the expression of OGT in the brain tissue of each group. **B** Escape latency and step-down tests were used to observe the changes of nerve function in CKD mice. **C** Immunofluorescence was used to detect the number of neurons in brain tissues of each group. **D** TUNEL staining was used to detect neuronal apoptosis. **E** Western blot analysis was used to detect the expression of EZH2, KLF2, CXCL1, and apoptosis-related proteins. *p < 0.05. vs. the sh-NC group (mice harboring neurons treated with sh-NC). *n* = 8 mice. The experiment was repeated 3 times independently.

We further verified whether OGT silencing can reduce nerve injury in CKD mice by injecting adenovirus into the CKD mouse model. Western blot analysis showed that the protein expression of OGT in brain tissues in response to sh-OGT was decreased (Fig. 6A). The results showed that the learning ability of mice in response to sh-OGT was improved (Fig. 6B). Immunofluorescence assay also showed that the number of neurons in brain tissues in response to sh-OGT was elevated (Fig. 6C). TUNEL staining showed that the apoptosis of neurons in response to sh-OGT was diminished (Fig. 6D, *p* < 0.05). Moreover, the expression of EZH2 and CXCL1 in brain tissues of mice in response to sh-OGT was decreased, the expression of KLF2 was increased, and the expression of Bax and cleaved-caspase3 was decreased while Bcl-2 expression was increased and no significant difference was witnessed regarding caspase3 expression (Fig. 6E). These results suggest that silencing OGT in vivo can reduce nerve injury in CKD mice.

## Discussion

Renal failure can cause frequent readmissions of their patients and is related to morbidity, mortality, and economic burden [18]. In the current study, we explored the regulatory mechanism of OGT in hypercalcemia-induced nerve injury in renal failure and found that OGT promotes this injury by regulating the EZH2/KLF2/CXCL1 axis.

Initially, we found in this study that downregulation of OGT reduced the toxicity of primary neurons induced by hypercalcemia by inhibiting the expression of EZH2. As previously reported, OGTase’s p78 subunit together with elevated protein and activity for OGTase was found in hyperglycemic rat aortic smooth muscle cells, and the aberrant O-GlcNAc modification of intracellular proteins is responsible for glucose toxicity to vascular tissues and thus involved in hyperglycemia which may result in renal failure [19]. Moreover, OGT was revealed to participate in the regulation of nerve injury response in the neuropathy related to diabetes and aging [20]. Strikingly, the interaction between OGT and EZH2 has been previously identified. For instance, it was found that OGT in the dorsal area CA1 of the hippocampus could play a control role over epigenetic modulation through EZH2/H3K27me3 during memory consolidation [7]. Additionally, microRNA-101-regulated OGT was found to promote EZH2 protein stability in colorectal cancer [21]. Moreover, OGT could regulate transcriptional repression of interleukin-15 in a muscle through EZH2 [22]. EZH2 antagonized by Wilm’s tumor 1 could impair renal function while increasing podocyte injury in diabetic rats and patients with diabetic nephropathy [23]. Increased levels of EZH2 were found in the spinal dorsal horn in rats with neuropathic pain following nerve injuries and EZH2 could affect spinal neuroinflammation [9].

Our mechanistic study further revealed that EZH2 could promote the expression of CXCL1 in primary neurons by inhibiting KLF2, thereby promoting the toxicity of primary neurons induced by hypercalcemia. Intriguingly, mounting evidence has demonstrated the regulatory relationship between EZH2 and KLF2 in a variety of cancers. For example, recruited EZH2 by long non-coding RNA (lncRNA) LINP1 could contribute to reduced expression of KLF2 in cervical cancer [10]. EZH2, interacting with lncRNA X-inactive specific transcript, could lead to suppression of KLF2 transcription in non-small cell lung cancer [11]. Moreover, EZH2 in interaction with LINC00702, could suppress the transcription of KLF2 in ovarian cancer [24]. To our acknowledge, KLF2 has been increasingly reported as a gene to alleviate kidney or neuron-related injury. Decreased KLF2 was reported to increase glomerular endothelial cell injury as well as kidney disease in a mouse model with unilateral nephrectomy [25]. In addition, downregulation of KLF2 by microRNA-25 was unfolded to aggravate hippocampal neuron injury induced by Amyloid-β1-42 via the Nrf2 signaling pathway in a mouse model with Alzheimer’s disease [26]. Of note, a previous study found that upregulation of CXCL1 was observed in peritoneal macrophages isolated from myeKLF2^-/-^ mice [14]. Downregulated CXCL1 in the presence of cortical neuron-derived exosomal microRNA-181c-3p could suppress neuroinflammation in astrocytes of rats with ischemic brain injury [27]. In addition, overexpression of renal level of CXCL1 was found to worsen renal function in wild-type mice, which could aid in exacerbated inflammation following renal ischemia-reperfusion injury, a key reason for intrinsic acute renal failure [15].

**Fig. 7:**
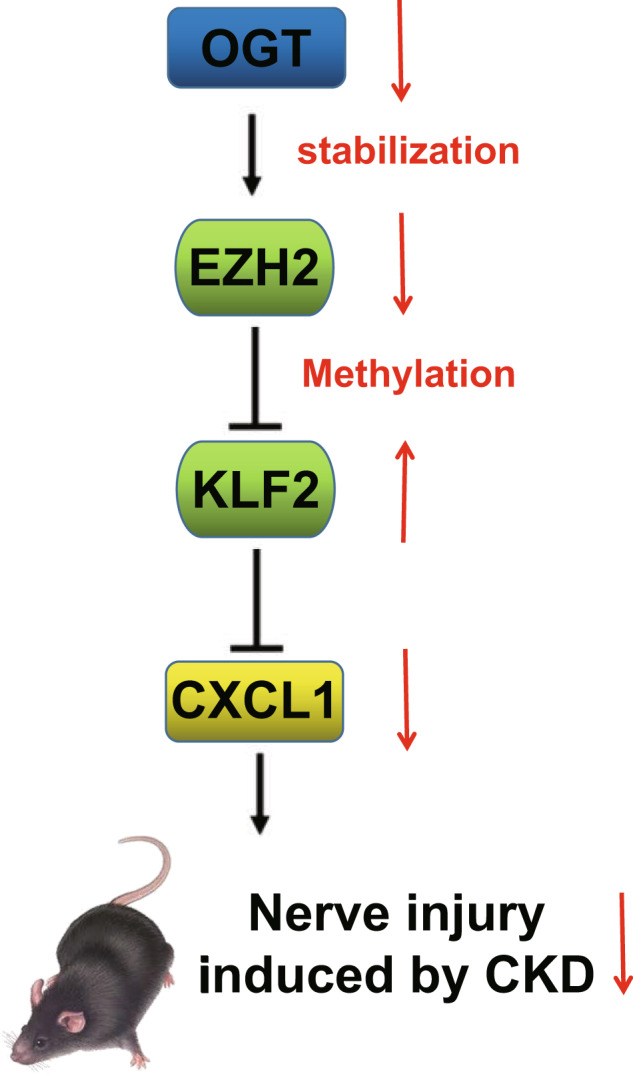
The molecular mechanism plot for the role of OGT in hypercalcemia-induced nerve injury in renal failure. Silencing OGT may inhibit the expression of EZH2, thereby upregulating KLF2 and then downregulating the expression of CXCL1, which inhibits hypercalcemia-induced nerve injury in renal failure.

To sum up, the present study demonstrates that silencing OGT decreases the expression of histone methylase EZH2, thereby upregulating KLF2 and then downregulating the expression of CXCL1, which alleviates hypercalcemia-induced nerve injury in renal failure (Fig. 7). This finding may provide a novel direction for the treatment of hypercalcemia-induced nerve injury in renal failure but still needs further verification.

## Supplementary information

supplementary information

Supplementary material

## Data Availability

The data and materials of the study can be obtained from the corresponding author upon request.
