# Supplementary material for: Silencing of O-linked N-acetylglucosamine transferase ameliorates hypercalcemia-induced neurotoxicity in renal failure by regulating EZH2/KLF2/CXCL1 axis
Source: Cell Death Dis. 2021 Aug 30;12(9):819. doi: 10.1038/s41419-021-04022-x (PMC8405781; doi:10.1038/s41419-021-04022-x)
Supplement: Supplementary file 1 — supplementary information [file 41419_2021_4022_MOESM1_ESM.docx]

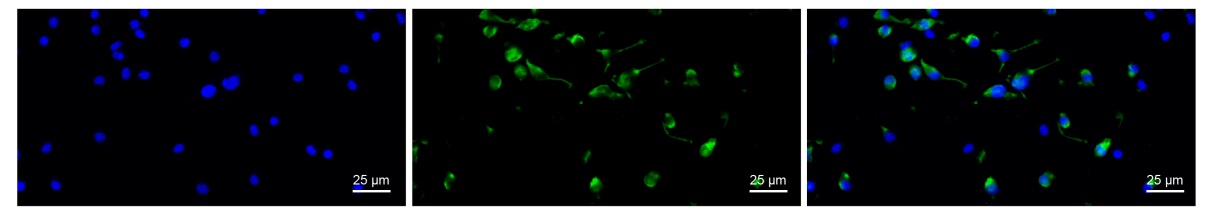


**Fig. S1.** NSE immunofluorescence staining image of hippocampal neurons. Left, DAPI-stained nuclei. Middle, NSE immunofluorescence staining image. Right, Merged image.


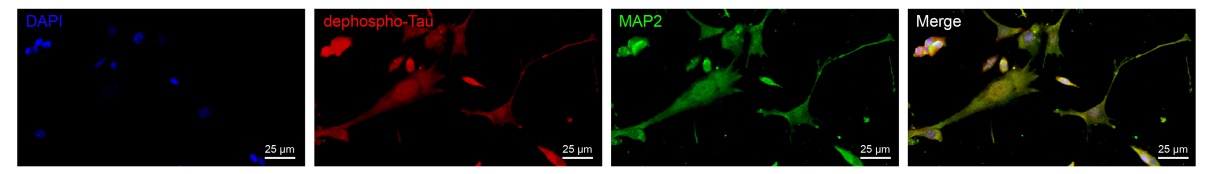


**Fig. S2.** Immunofluorescence images of hippocampal neurons for dephospho-Tau and MAP2.

**Supplementary table 1** Primer sequences for RT-qPCR

| Gene | Sequence |
| --- | --- |
| EZH2 | Forward: 5’-CTGCTCTGAATGGCAACTCC-3’ |
|  | Reverse: 5’-TTATTCATAGAGCCACCTGG-3’ |
| KLF2 | Forward: 5’-GAGCCTATCTTGCCGTCCTTT-3’ |
|  | Reverse: 5’-CACGTTGTTTAGGTCCTCATCC-3’ |
| GAPDH | Forward: 5’-GGTGAAGGTCGGTGTGAACG-3’ |
|  | Reverse: 5’-CTCGCTCCTGGAAGATGGTG-3’ |

Note: EZH2, enhancer of zeste homolog 2; KLF2, Krüppel-like factor 2; GAPDH, glyceraldehyde-3-phosphate dehydrogenase; RT-qPCR, reverse transcription-quantitative polymerase chain reaction
